# Social inequalities in mental and physical health derived from the COVID-19 pandemic in Spain beyond SARS-CoV-2 infection

**DOI:** 10.1186/s12939-023-01933-3

**Published:** 2023-07-24

**Authors:** Isabel Moreira, Montse Ferrer, Gemma Vilagut, Philippe Mortier, Mireia Felez-Nobrega, Joan Domènech-Abella, Josep-Maria Haro, Jordi Alonso

**Affiliations:** 1 Health Services Research Group, Hospital del Mar Research Institute, Doctor Aiguader 88, office 144, Barcelona, 08003 Spain; 2 Preventive Medicine and Public Health Training Unit PSMar-UPF-ASPB (Parc de Salut Mar – Universitat Pompeu Fabra - Agència de Salut Pública de Barcelona), Barcelona, Spain; 3 CIBER de Epidemiología y Salud Pública (CIBERESP), Barcelona, Spain; 4 Department of Experimental and Health Sciences, Universitat Pompeu Fabra, Barcelona, Spain; 5 Parc Sanitari Sant Joan de Déu, Barcelona, Spain; 6 CIBER de Salud Mental (CIBERSAM), Madrid, Spain; 7 Instituto de Investigación del Hospital de la Princesa, Madrid, Spain

**Keywords:** EQ-5D-5L, Health inequalities, COVID-19, Indirect impact

## Abstract

**Background:**

Understanding the impact of the COVID-19 crisis on health involves conducting longitudinal studies to evaluate the inequalities that may have been exacerbated by the pandemic. The purpose of this study was to estimate differences in physical and mental health derived from the COVID-19 pandemic, beyond SARS-CoV-2 infection, in the Spanish general population according to the participants’ level of education; and to assess the evolution of these differences from June 2020 (just after the lockdown) to nine months later (February-March 2021).

**Methods:**

This is a longitudinal prospective study of a representative sample of non-institutionalized Spanish adults, through computer-assisted telephone interviews. Mobility, self-care, usual activities, pain/discomfort and anxiety/depression problems were measured with EQ-5D-5L. Prevalence ratio (PR) between high and low education levels and adjusted PR were estimated by Poisson regression models. Analyses were stratified by gender.

**Results:**

A total of 2,000 participants answered both surveys. Individuals with low level of education reported more health problems in both genders, and absolute inequalities remained quite constant (mobility and self-care problems) or decreased (pain/discomfort and anxiety/depression problems). The greatest relative inequalities were observed just after the lockdown, with age-adjusted PR ranging from 1.31 (95%CI 1.08–1.59) for women and 1.34 (95%CI 1.05–1.69) for men in pain/discomfort to 2.59 (95%CI 0.98–6.81) for women and 4.03 (95%CI 1.52–10.70) for men in self-care; aPR decreased after nine months for most dimensions.

**Conclusions:**

Prevalence of health problems increased during the COVID-19 pandemic in all education groups, but the increase was higher in women and men with a high level of education, suggesting that its impact appeared later in this group. Further analysis on the role of governmental economic aid given to vulnerable people might shed light on this evolution.

**Supplementary Information:**

The online version contains supplementary material available at 10.1186/s12939-023-01933-3.

## Background

The COVID-19 lockdown in Spain lasted from March 14th to May 4th 2020, with limited mobility, home confinement, telecommuting and online teaching. The situation was similar to that of neighbouring countries such as France [1] and Italy [2]. Numerous studies have been published on the changing health-related behaviours in the general population during this phase of the State of Alarm (physical exercise, diet, alcohol and tobacco consumption), suggesting a negative impact in most of them [3] and a widening of inequalities [4].

In Spain, one of the countries most affected by the pandemic in the first waves, there was a decrease in life expectancy that was among the highest recorded in middle- and high-income countries by 2020: -1.27 (-1.57 to -0.99), similar to the global decrease observed in Italy (-1.35; 95CI% -1.72 to -0.99) and higher than in France (-0.67; 95%CI -0.87 to -0.51) [5]. Part of these excess deaths are indirectly caused by the situation created by the pandemic, as found also in other countries [6, 7]. Since the beginning of the State of Alarm in Spain [8], non-COVID-19 and non-urgent hospital health services were practically paralyzed and the population feared going to hospital due to the risk of infection, two circumstances which have generated unmet needs in health [9–11]. A similar situation was described in Italy, where inequalities in access to healthcare services increased during the pandemic compared to the pre-pandemic period [2], and where mortality decreased in the second wave [12], similarly to what was reported in Spain [13].

Conceptual models of health inequalities developed in the context of the COVID-19 pandemic theorize that the government’s measures to protect the population from the health crisis, collectively known as confinement, can especially affect the most vulnerable populations [14, 15]: they could accentuate previously existing health inequalities, but also create new ones, and not only because of epidemiological vulnerability to the virus. Each axis of inequality interacts with each other, and can worsen and be worsened by others if mitigation strategies are not applied. Recent evidence suggests that the Governments’ financial support during COVID-19 pandemic had a protective influence in the health of the population who benefited from the aids [16, 17].

So far, most of the studies on inequalities and COVID-19 have focused on risk of infection and mortality both in Spain [18–20] and internationally [21–23], while studies on the inequalities in the pandemic’s indirect impact on the health of the population are scarce, and addressed mainly to mental health [24, 25]. Cross-sectional studies focused on health inequalities measured with the EQ-5D-5L were performed in few countries during the COVID-19 pandemic [17, 26–29]. A Moroccan study [26] showed a greater negative impact on health of widows and the socioeconomically more vulnerable groups. A study carried out in Portugal [27] revealed a greater impact on women, especially in the anxiety/depression dimension. In Japan [17], a positive impact on health was observed in the population that benefited from universal financial support. A population health survey in US [28] found younger people and those with lower income to have worse health outcomes. Finally, a study evaluating the impact of the Government’s response stringency in eight countries [29] showed that it was inversely related to health.

Understanding the impact of the COVID-19 crisis on health involves conducting longitudinal studies to evaluate the inequalities that may have been exacerbated by the pandemic. Therefore, our aim was to estimate differences in physical and mental health derived from the COVID-19 pandemic, beyond SARS-CoV-2 infection, in the Spanish general population according to the participants’ level of education; to assess the evolution of these differences from June 2020 (just after the lockdown) to nine months later (at the end of the third wave of the pandemic); and to identify factors that could explain health inequalities in the aforementioned period.

## Methods

### Study design, population and sampling

This is a longitudinal prospective study using data from a general population sample, as part of the MIND COVID Project [30]. Participants were interviewed twice, first in June 2020, at the end of lockdown in Spain, and then nine months later in February-March 2021. Reporting of this longitudinal prospective study follows the STROBE criteria (https://www.strobe-statement.org/), and the checklist can be found in the Supplementary Material.

The target population was non-institutionalized Spanish adults (≥ 18 years) without language barriers. A company specialized in survey research services (IPSOS) carried out the sample selection and the computer-assisted telephone interviews. The generation of the sample has been described in previous publications [31]. The sample was drawn through a dual-frame random digital dialling telephone survey, including both landlines and mobile telephones. First, a sample of Spanish mobile telephone numbers was generated through an automated system, which avoids duplicates with other projects. Subsequently, landline numbers were selected from an internal database developed and maintained by the survey company to ensure that all Spanish geographical areas were correctly represented. Up to seven calls at different times of the day and varied days of the week were attempted to each number.

The distribution of the participants was planned according to quotas proportional to the Spanish population in terms of age groups, sex and autonomous community, according to data from July 2019 of the National Institute of Statistics in Spain [32]. Of a total of 138,656 phone numbers sampled, 45,002 were classified as non-eligible (i.e., 43,120 numbers no longer existed, 984 were business numbers, 444 belonged to persons with Spanish language barriers, 268 were fax numbers, and 186 belonged to the quota that was already completed), and 72,428 had unknown eligibility (i.e., no contact was made after the seven attempted calls), resulting in a 16,5% cooperation rate (i.e., the proportion of all cases interviewed of all eligible units ever contacted). Finally, to achieve the target size of 3,500 participants, 5 or 6 replacements were needed for every person contacted: 75% were contacted by mobile and 25% by landline telephones (n = 2,649 and n = 851, respectively). More details about the limitations of the data used can be found in the Limitations section.

The initial interview was conducted on a sample of 3,500 individuals, and the follow-up interview was carried on a subsample of 2,000 individuals out of the initial respondents (60% of the baseline sample), due to budgetary issues.

### Variables and measurement instruments

#### The EQ-5D-5L

The EQ-5D-5L covers five dimensions of health (mobility, self-care, usual activities, pain/discomfort, and anxiety/depression) with five levels of severity, from none to extreme problems. The number of unique health states it describes is 3125 (5^5^). Validity has been demonstrated extensively in general population, also for the Spanish version of the instrument [33, 34].

#### Socio-demographic data

Gender (male; female), age, country of birth, education, and income, among others, were recorded. Level of education was recorded following the International Standard Classification of Education (ISCED) 2011 categories [35]: 0–1) Primary education or not completed; 2) Secondary education with graduate degree; 3) Baccalaureate and similar; 4–5) Undergraduate vocational training courses; 6) Bachelor’s or equivalent university degree; and 7–8) Master’s, postgraduate or doctoral degrees. These categories were grouped into “low” (Secondary education or lower), “middle” (Baccalaureate and undergraduate) or “high” education (University degree or higher).

Information about income was collected through the question “*Before the arrival of the COVID-19 pandemic to Spain, which of the following better represents the net monthly income of your home, after tax, Social Security, etc. deductions?”* with 12 response options which were grouped into three: < 1050€; 1050 to < 2700€; ≥ 2700€.

#### Chronic health conditions

Participants were asked “*Do you currently have any of the following chronic health conditions: respiratory disease; cardiovascular problems; diabetes; cancer; chronic liver disease; immune problems; or other?* A summary indicator based on the number of reported chronic conditions was categorized into four groups: none, 1, 2, 3 or more chronic conditions.

#### COVID-19 and lockdown variables

History of SARS-CoV-2 past infection in oneself or in those closest to them was asked through questions about diagnosis and severity (no infection; mild; severe). Details asked about life while the lockdown was in force were: characteristics of the household members (living with a partner; children in care; elderly or disabled persons in care) and house conditions (number of bathrooms and bedrooms, and access to a balcony or private garden). Participants were asked about their working status, and those who were employed were asked “*Is your job considered essential?” and “How often do you work outside the home?”*

#### Statistical power

The sample size was calculated to achieve the primary objectives of the MIND COVID study, focused on mental health, with an estimated prevalence of mental health problems of 20%. The statistical power of the 2,000 participants with follow-up is of at least 80% at a significance level of 5%, to detect as statistically significant prevalence ratios ≥ 1.75 in the EQ-5D-5L dimensions between extreme groups of education, separately in women and men.

### Statistical analysis

Analyses were stratified by gender and interview time (after the lockdown in June 2020, and nine months later in February-March 2021), and were done with the subsample of participants who completed both interviews. To ensure sample representativeness and to compensate for potential survey non-response bias, all data was weighted with post-stratification weights to match the sample to the distribution of the adult general population of Spain according to age groups, sex and geographic region. Missing data were minimal (median 0.17% [IQR 0.06–0.59%]), and a complete case analysis was carried out.

Distribution of study variables was reported as unweighted frequencies and weighted percentages. Differences by gender and by completion of the second interview were tested using χ2. Our primary explanatory variable of inequalities was education level.

The five-level response scale of each EQ-5D-5L dimension was dichotomized into “no problems” versus “any problem” (slight, moderate, severe, or extreme problems) and prevalence of any problem in each dimension was estimated for both interviews. Inequalities in the EQ-5D-5L dimensions by education level were assessed by quantifying the prevalence difference and the prevalence ratio between extreme groups of education level (i.e., high and low education level), which was used as the main explanatory variable. Crude and age-adjusted prevalence ratios by education level and corresponding 95% CI (high education as reference category) were calculated with Poisson regression models, with robust error variance estimation [36] for each EQ-5D-5L dimension as dependent variable. A sensitivity analysis was done using declared monthly income as the main explanatory variable instead of education level, to compare results obtained with both indicators of socioeconomic position.

Risk and protective factors that could be associated to health inequalities during the COVID-19 pandemic were added to the Poisson regression models to adjust by them. The independent variables included in these models, selected from conceptual theoretical models [14, 37], were: education, age, number of chronic health conditions, history of SARS-CoV-2 past infection, lockdown social variables, working status and conditions. Country of birth, despite being one of the main axes of inequalities, was not included in the models as it was neither statistically significant in any model, nor the rest of variables changed with its inclusion, therefore we selected the simpler models. This was probably explained by the need of grouping middle and low-income countries into a single category due to the small sample size.

The level of statistical significance was set to α = 0,05. The statistical analysis was carried out using the R software version 1.4.1106 [38].

## Results

Among the 2,000 participants answering both initial and follow-up interviews, statistically significant differences between women and men were found in almost all the socioeconomic variables and working conditions *(*Table 1*)*: Women reported significantly worse socioeconomic position (education level and income), lower rates of employment, lower frequency of working outside the home, and feeling more frequently unprotected against SARS-CoV-2 at their work. When comparing the sample of participants who answered both surveys (n = 2,000) and those who only answered the first survey (n = 1,500), the latter had a slightly higher proportion of younger people, of people declaring not having University studies (55.65% vs. 65.31%) and having an income below 1050 € (19.71% vs. 15.58%) (see Supplementary Table S1).

**Table 1:** Socio-demographic differences between women and men who answered both interviews, at the end of the lockdown in June 2020 and nine months later in February-March 2021. Unweighted frequencies and weighted percentages

	Women	Men	Weighted p-value
**SOCIODEMOGRAPHIC**			
**Age group**			0.087
18–34 years	194 (21.17%)	171 (23.09%)	
35–54 years	492 (36.99%)	377 (39.59%)	
55–64 years	249 (15.82%)	181 (16.08%)	
65 years and over	175 (26.02%)	161 (21.24%)	
**Education level**			0.005*
Secondary education or lower	262 (25.15%)	171 (19.26%)	
Baccalaureate to undergraduate	364 (31.60%)	315 (35.49%)	
University degree or higher	483 (43.25%)	403 (45.25%)	
*Missings*	*1 (0.09%)*	*1 (0.11%)*	
**Country of birth**			0.166
Spain	969 (87.79%)	799 (89.75%)	
Abroad	141 (12.21%)	91 (10.25%)	
**Country of birth if born abroad**			0.443
Low income countries	24 (18.34%)	12 (12.85%)	
Middle income countries	91 (62.67%)	58 (63.48%)	
High income countries	25 (18.99%)	21 (23.66%)	
*Missings*	*1 (0.09%)*	*0 (0%)*	
**Monthly income**			< 0.001*
< 1050 €	271 (27.80%)	121 (14.33%)	
1050 to < 2700 €	550 (54.49%)	479 (57.57%)	
≥ 2700 €	188 (17.71%)	245 (28.09%)	
*Missings*	*101 (9.01%)*	*45 (5.06%)*	
**NUMBER OF CHRONIC HEALTH CONDITIONS**			0.113
0	669 (59.04%)	539 (60.78%)	
1	302 (27.71%)	261 (29.23%)	
2	109 (10.51%)	65 (7.43%)	
3 or more	29 (2.73%)	24 (2.56%)	
*Missings*	*1 (0.09%)*	*1 (0.11%)*	
**COVID AND LOCKDOWN**			
**COVID-19: Personal infection**			0.192
Negative	1074 (97.24%)	865 (97.45%)	
Positive or COVID diagnosis, non-severe	32 (2.76%)	21 (2.28%)	
Hospitalized	0 (0%)	3 (0.27%)	
*Missings*	*4 (0.36%)*	*1 (0.11%)*	
**COVID-19: Having a beloved one infected**			0.098
No	591 (54.71%)	436 (49.98%)	
Yes, but not inner circle	470 (41.69%)	409 (46.44%)	
Yes, inner circle	40 (3.60%)	33 (3.58%)	
*Missings*	*9 (0.81%)*	*12 (1.35%)*	
**Living with a partner**			0.045*
No	387 (37.94%)	285 (33.65%)	
Yes	723 (62.06%)	605 (66.35%)	
**Having children in care**			0.915
No	718 (69.22%)	601 (69.44%)	
Yes	392 (30.78%)	289 (30.56%)	
**Having elderly people or people with a disability in care**			0.130
No	934 (83.58%)	771 (86.01%)	
Yes	176 (16.42%)	119 (13.99%)	
**Number of bedrooms**			0.583
0 to 3 bedrooms	732 (65.35%)	592 (66.51%)	
More than 3 bedrooms	374 (34.65%)	296 (33.49%)	
*Missings*	*4 (0.36%)*	*2 (0.22%)*	
**Access to balcony or private garden**			0.100
No	296 (26.62%)	271 (29.94%)	
Yes	813 (73.38%)	618 (70.06%)	
*Missings*	*1 (0.09%)*	*1 (0.11%)*	
**Working status**			< 0.001*
Working	506 (39.93%)	501 (54.35%)	
Working but sick leave	39 (3.22%)	15 (1.54%)	
Unemployed	213 (17.59%)	124 (14.03%)	
Homemaker	95 (8.67%)	4 (0.39%)	
Student	41 (4.47%)	32 (4.35%)	
Disabled	31 (2.52%)	23 (2.25%)	
Retired	171 (22.70%)	186 (23.00%)	
Other	10 (0.90%)	1 (0.08%)	
*Missings*	*4 (0.36%)*	*4 (0.45%)*	
**Working conditions (only for workers)**	**N = 506**	** N = 501**	
**Frequency of working outside home**			0.003*
Never	240 (47.92%)	192 (38.78%)	
Rarely	50 (9.92%)	69 (13.48%)	
Sometimes	34 (6.38%)	42 (8.33%)	
Frequently	11 (2.01%)	22 (4.18%)	
Usually	26 (4.92%)	42 (8.74%)	
Always	144 (28.84%)	133 (26.50%)	
*Missings*	*1 (0.20%)*	*1 (0.20%)*	
**Job considered as essential**			0.602
Yes	269 (53.77%)	259 (52.09%)	
No	226 (46.23%)	232 (47.91%)	
*Missings*	*11 (2.17%)*	*10 (2.00%)*	
**Frequency of feeling unprotected against SARS-CoV-2 at work**			< 0.001*
Never	270 (52.70%)	289 (57.07%)	
Rarely	59 (11.90%)	75 (15.13%)	
Sometimes	67 (12.55%)	74 (14.95%)	
Usually	28 (6.13%)	25 (5.27%)	
Always	79 (16.41%)	32 (6.62%)	
Doesn’t know	2 (0.32%)	5 (0.96%)	
*Missings*	*1 (0.20%)*	*1 (0.20%)*	

**Fig. 1:**
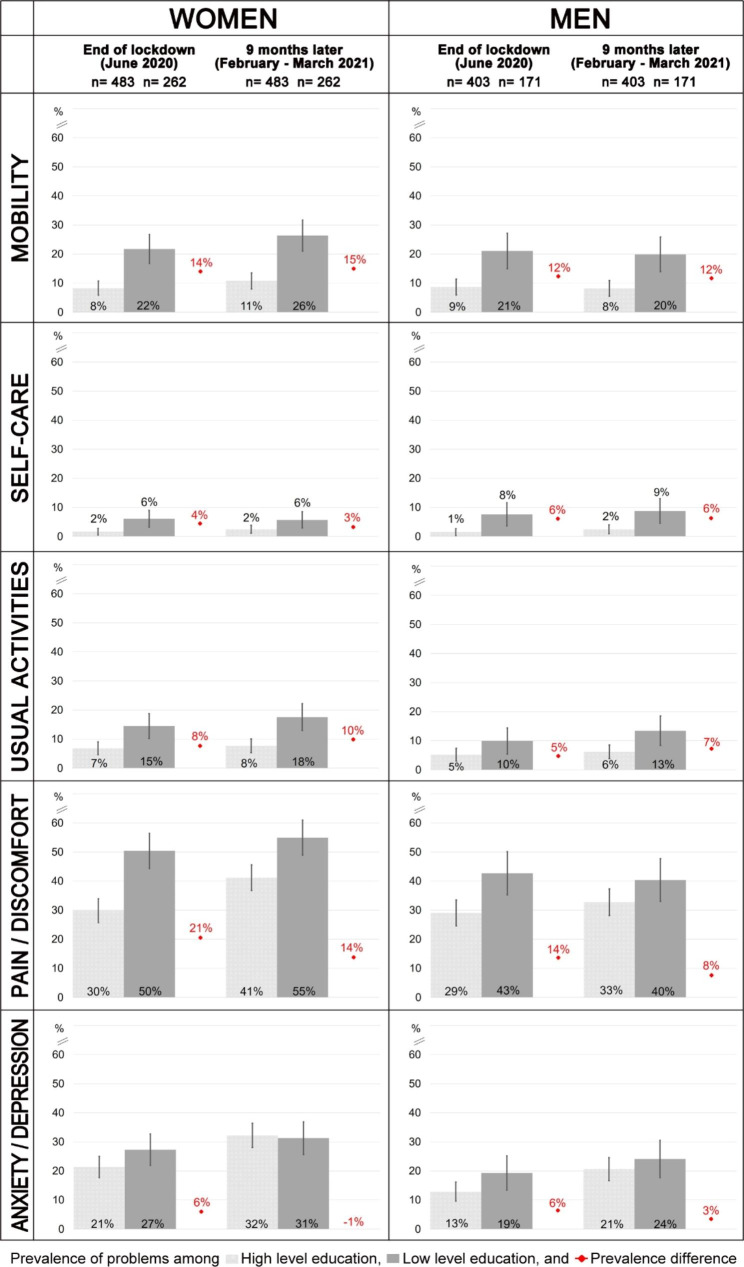
Unweighted prevalence (and 95% confidence intervals) of health problems reported in each EQ-5D-5L dimension, stratified by gender. Participants with a high level of education are represented in the light grey bar, while those with a low level of education are shown in dark grey

Figure 1 shows unweighted prevalence of problems measured with EQ-5D-5L in extreme education levels (represented in bars, with light grey for higher and dark grey for lower level of education). Results of the first interview are on the left and results of the second interview are on the right of every box. Prevalence of problems was higher among women. Both women and men with high education level presented in general lower prevalence of problems than those with low education level. The highest prevalence of problems was observed in pain/discomfort and anxiety/depression dimensions. Prevalence differences after lockdown between extreme groups of education level among women ranged from 21% in pain/discomfort to 4% in self-care and, among men, from 14% in pain/discomfort to 5% in usual activities. Nine months later, prevalence differences in mobility and self-care remained similar, and decreased in pain/discomfort (from 21% to 14% in women and from 14% to 8% in men) and anxiety/depression (from 6% to -1% in women and from 6% to 3% in men), mainly due to an increase in the prevalence of problems in participants with a high level of education.

**Fig. 2:**
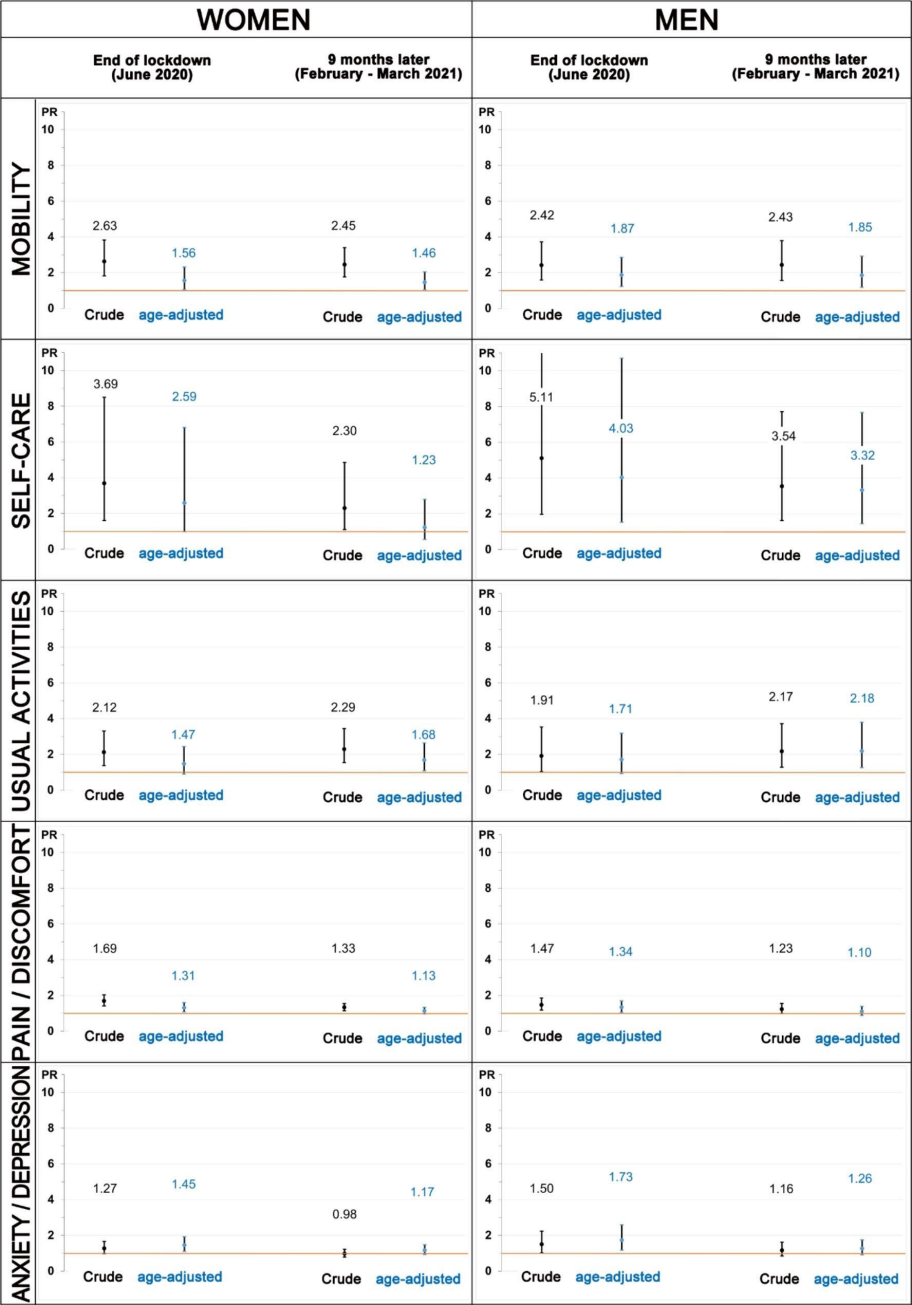
Crude and age-adjusted prevalence ratios (and 95% confidence intervals) of health problems reported in each EQ-5D-5L dimension among participants with a low level of education compared to those with a high level of education, stratified by gender

Weighted crude and age-adjusted prevalence ratios (PR) are shown in Fig. 2. Most crude and age-adjusted PR were statistically significant. A decreasing trend during follow-up is observed in the age-adjusted PR between extreme education level groups, except for the usual activities dimension. For example, anxiety/depression problems in women decreased from 1.45 to 1.17, and from 1.73 to 1.26 in men. Supplementary figure S1 shows the evolution of these inequalities among the working participants, with similar patterns to the ones found in the global sample, except for usual activities in both genders and self-care in men.

**Fig. 3:**
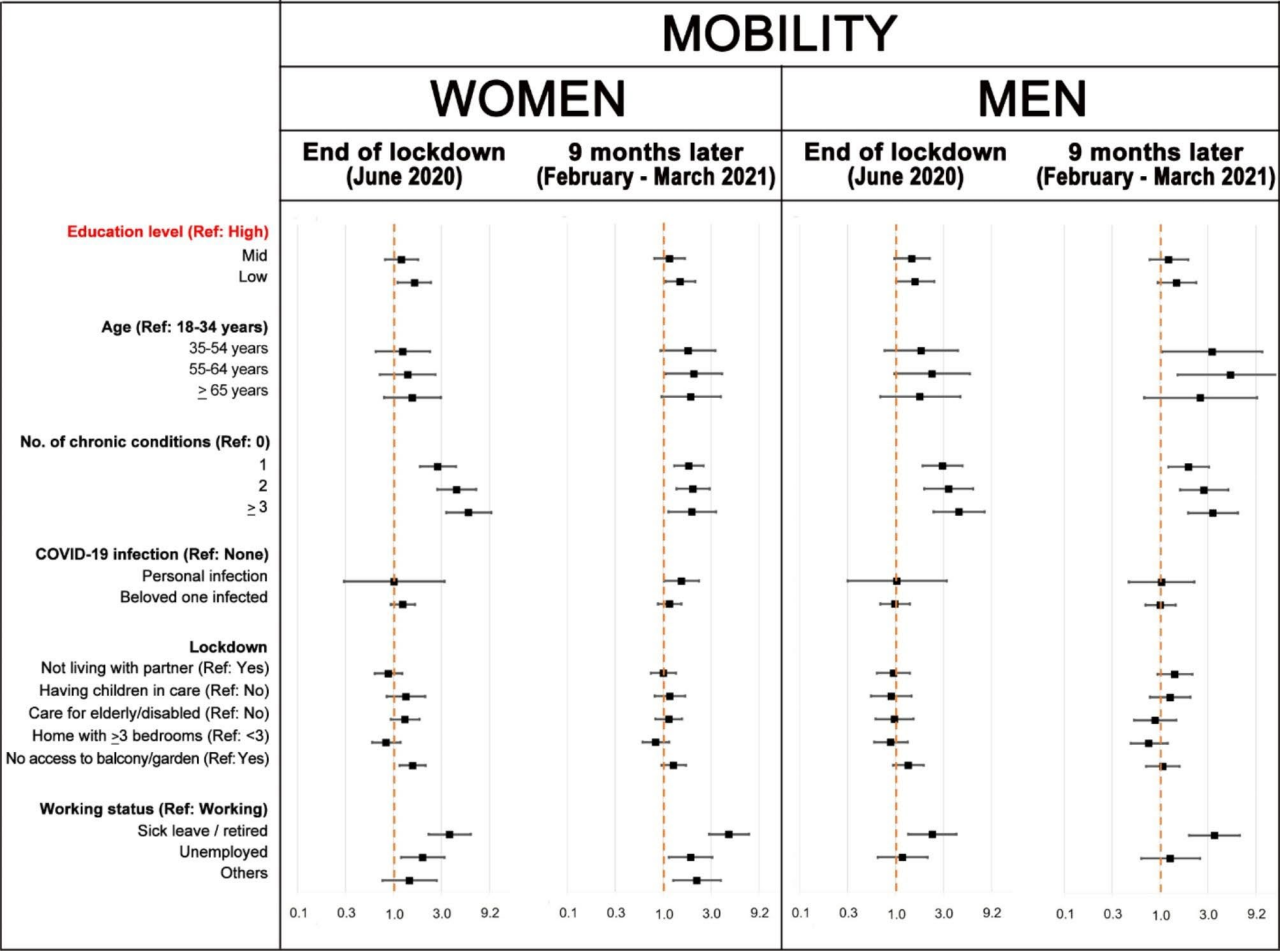
Poisson regression models of having mobility problems: adjusted prevalence ratios (aPR) and 95% confidence intervals compared to the reference (Ref) category. The aPR for the category ‘other working status’ in men could not be estimated due to low numbers

Figure 3 shows Poisson regression models for mobility problems just after the lockdown (June 2020) and nine months later (February-March 2021). Just after the lockdown, women with low education level presented significantly higher prevalence of mobility problems than those with high education level after adjusting for all the explanatory variables included in the model (aPR = 1.58; 95%CI 1.06–2.33). Also, number of chronic health conditions, access to a garden or balcony (green spaces), and working status were significantly associated with mobility problems in this first interview, but nine months later, education level and access to green spaces were no longer significantly associated. In men, only number of chronic health conditions was significantly associated in both models for mobility.

**Fig. 4:**
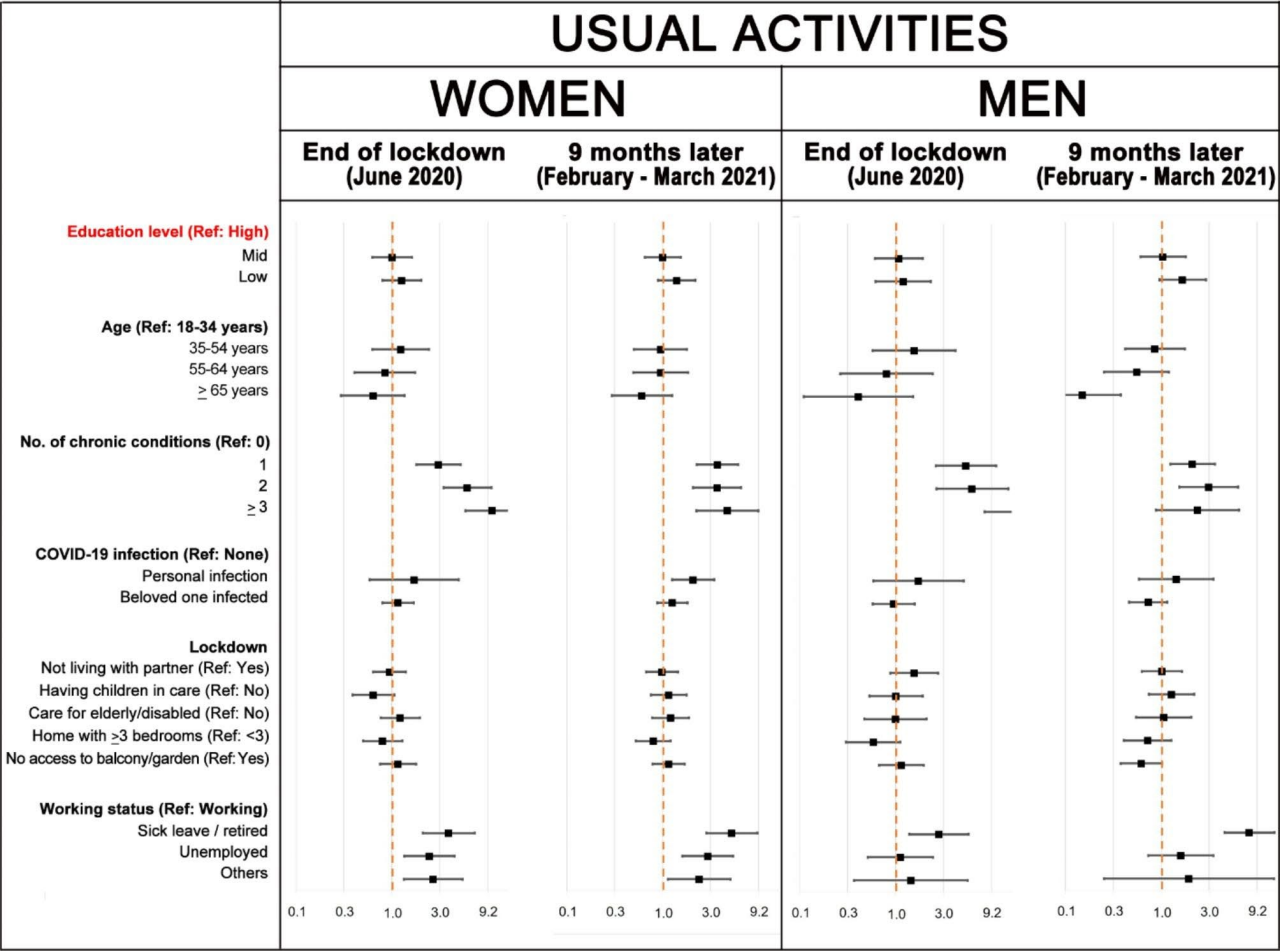
Poisson regression models of having problems in usual activities: adjusted prevalence ratios and 95% confidence intervals compared to the reference (Ref) category

**Fig. 5:**
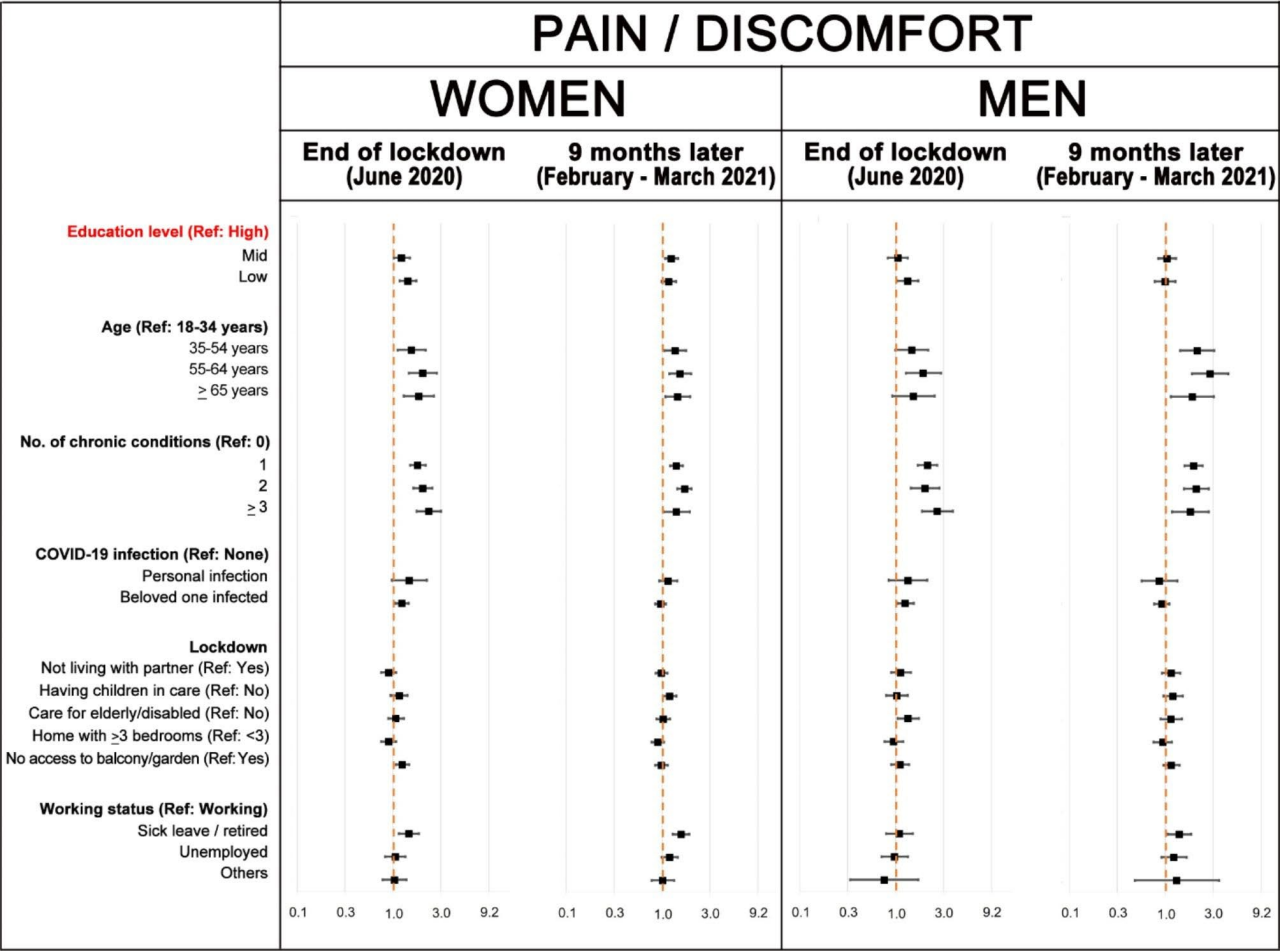
Poisson regression models of problems regarding having pain / discomfort: adjusted prevalence ratios and 95% confidence intervals compared to the reference (Ref) category

**Fig. 6:**
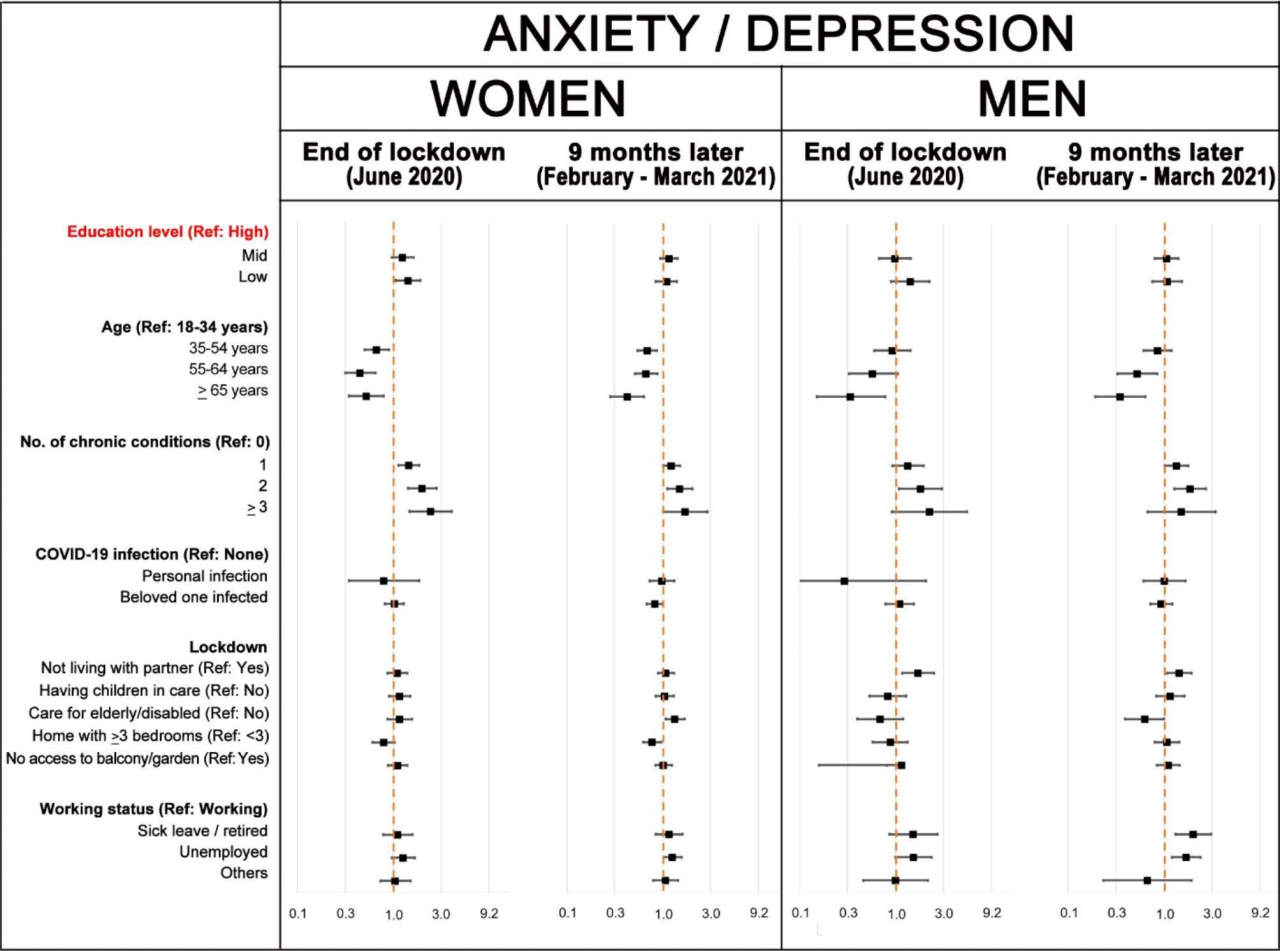
Poisson regression models of having anxiety / depression problems: adjusted prevalence ratios and 95% confidence intervals compared to the reference (Ref) category

Figures 4, 5 and 6 show that, in general, age and number of chronic health conditions were significantly and consistently associated with usual activities, pain/discomfort and anxiety/depression EQ-5D dimensions. Association with education level only remains significant at both interviews in pain/discomfort among women. Furthermore, education level was significant for anxiety/depression problems in women at the first interview and for usual activity problems in men at the second interview. Having had COVID-19 or having a relative or beloved one who suffered the disease was significantly associated with health in some models. In February-March 2021, new variables became significantly associated with health in women: taking care of children or of elderly/ disabled persons increased the risk of pain/discomfort and anxiety/depression problems, respectively; and the number of bedrooms at home decreased the risk of problems in both dimensions. Finally, men not living with a partner had a significantly higher prevalence of anxiety/depression problems in both interviews. As the number of people with self-care problems was very small, the precision of estimators of this dimension is very low and, therefore, results of the multivariate models were not included in the article (see Supplementary figure S2).

The sensitivity analysis performed with income level (Table 2) shows a similar pattern of inequalities. Using income as the main explanatory variable instead of educational level, the magnitude of the aPR decreased and was no longer significant in some models among women, but it remained very similar in most models among men, probably due to the higher number of missings among women than men (9.01% vs. 5.06%, respectively). For example, in pain/discomfort problems, aPR for low education level was 1.37 (1.12–1.67) while aPR for low income was 1.18 (0.89–1.56) in women in 2020. In men, the aPR obtained with education and income level were very similar, both in 2020 (aPR 1.28 or 1.40) and in 2021 (aPR 0.96 or 0.98).

**Table 2:** Adjusted Prevalence Ratios (aPR, and 95% confidence intervals) of having health problems in each EQ-5D-5L dimension according to education level (principal analysis) or income level (sensitivity analysis). Poisson regression models adjusted by age, number of chronic health conditions, history of SARS-CoV-2 past infection, lockdown social variables, and working status

Variables	WOMEN				MEN			
	June 2020		March 2021		June 2020		March 2021	
	aPR by education level	aPR by income level	aPR by education level	aPR by income level	aPR by education level	aPR by income level	aPR by education level	aPR by income level
**Mobility**								
Highest level	Ref.	Ref.	Ref.	Ref.	Ref.	Ref.	Ref.	Ref.
Intermediate level	1.16 (0.79–1.72)	1.32 (0.75–2.32)	1.12 (0.78–1.60)	1.05 (0.66–1.68)	1.42 (0.94–2.16)	0.95 (0.60–1.50)	1.18 (0.75–1.87)	1.13 (0.69–1.84)
Lowest level	1.58 (1.06–2.33)	1.39 (0.76–2.56)	1.44 (1.02–2.04)	1.08 (0.65–1.79)	1.53 (0.97–2.39)	1.39 (0.78–2.50)	1.43 (0.91–2.26)	1.73 (0.98–3.05)
**Self-care**								
Highest level	Ref.	Ref.	Ref.	Ref.	Ref.	Ref.	Ref.	Ref.
Intermediate level	1.38 (0.57–3.36)	0.91 (0.30–2.76)	1.21 (0.58–2.53)	1.34 (0.42–4.25)	0.93 (0.33–2.59)	0.53 (0.19–1.44)	0.87 (0.36–2.11)	0.92 (0.34–2.50)
Lowest level	1.80 (0.72–4.49)	1.17 (0.33–4.14)	1.20 (0.56–2.58)	1.05 (0.28–3.92)	2.68 (1.03–6.96)	1.53 (0.65–3.62)	1.92 (0.82–4.49)	2.05 (0.80–5.21)
**Usual Activities**								
Highest level	Ref.	Ref.	Ref.	Ref.	Ref.	Ref.	Ref.	Ref.
Intermediate level	0.97 (0.61–1.54)	1.21 (0.58–2.50)	0.96 (0.63–1.48)	1.27 (0.59–2.70)	1.04 (0.59–1.82)	1.61 (0.76–3.41)	1.00 (0.59–1.72)	1.00 (0.56–1.78)
Lowest level	1.22 (0.77–1.93)	1.23 (0.57–2.70)	1.34 (0.86–2.08)	1.27 (0.56–2.88)	1.15 (0.60–2.20)	2.75 (1.23–6.17)	1.59 (0.92–2.76)	1.16 (0.61–2.16)
**Pain / Discomfort**								
Highest level	Ref.	Ref.	Ref.	Ref.	Ref.	Ref.	Ref.	Ref.
Intermediate level	1.18 (0.98–1.43)	1.22 (0.95–1.56)	1.20 (1.03–1.40)	1.23 (0.99–1.53)	1.02 (0.80–1.28)	1.13 (0.87–1.46)	1.01 (0.82–1.24)	1.08 (0.86–1.36)
Lowest level	1.37 (1.12–1.67)	1.18 (0.89–1.56)	1.13 (0.95–1.34)	1.33 (1.05–1.69)	1.28 (0.99–1.64)	1.40 (1.00-1.97)	0.96 (0.75–1.22)	0.98 (0.72–1.35)
**Anxiety / Depression**								
Highest level	Ref.	Ref.	Ref.	Ref.	Ref.	Ref.	Ref.	Ref.
Intermediate level	1.20 (0.93–1.56)	1.01 (0.72–1.42)	1.12 (0.91–1.37)	0.78 (0.61–1.01)	0.95 (0.65–1.38)	1.26 (0.79–1.99)	1.02 (0.77–1.35)	1.31 (0.94–1.83)
Lowest level	1.37 (1.02–1.83)	1.04 (0.72–1.52)	1.06 (0.82–1.35)	0.83 (0.62–1.10)	1.36 (0.86–2.14)	1.14 (0.62–2.08)	1.04 (0.73–1.46)	1.08 (0.69–1.67)

Education levels: Highest (University degree or higher); Intermediate (Baccalaureate to undergraduate); and Lowest (Secondary education or lower)

Monthly income levels: Highest (≥ 2700 €); Intermediate (1050 to < 2700 €); and Lowest (< 1050 €)

## Discussion

### Main results

Using longitudinal prospective data of a representative sample of the general population in Spain, we estimated absolute and relative health inequalities by education level and protective and risk factors for these differences. According the EQ-5D-5L dimension, absolute inequalities in health remained quite constant (mobility and self-care problems) or decreased (pain/discomfort and anxiety/depression problems) in both genders. The greatest relative inequalities took place just after the lockdown in June 2020 and decreased nine months later in most dimensions, suggesting that health problems increased later in the high education level group. Age and chronic health conditions were highly associated to physical and mental health problems, explaining a great part of health inequities by education level, while certain factors related to housing during lockdown seem to have been protective.

### Health problems increased later in high education level group

The last National Health Survey in Spain with EQ-5D-5L measurements was carried out in 2011/12 [39], when the adjusted odds ratio of having no problems in each EQ-5D-5L dimension for persons with high education level (compared to low) ranged from 1.38 (95%CI 1.22, 1.55) in pain/discomfort to 1.72 (95%CI 1.46, 2.04) in mobility. In fact, very similar to the age-adjusted PR obtained in our study in 2020 just after the lockdown.

Contrary to our initial hypothesis, we observed a decrease in relative health inequalities during the pandemic. Bearing in mind the barriers to know whether our data largely differs with the data before the pandemic, the first possible scenario is that inequalities could have remained stable during the lockdown period if both educational groups were equally affected. Another possible scenario is that health inequalities could have increased during the strict lockdown, as socioeconomically vulnerable people would have suffered the most. This could explain that initial PR of problems found between extreme educational groups was very similar to the PR found a few years after the financial crisis of 2008 in Spain [39]. Regardless of what happened during the lockdown, there was a subsequent increase in health problems among participants with high level of education as the pandemic progressed. This way, as prevalence of problems grew more during the follow-up among the higher education level group, relative inequalities in health problems decreased.

Two studies have previously reported the protective role of the Government aids in Spain [16] and Japan [17]. The Spanish study [16], using data from bank records, revealed that economic inequalities would have increased a 30% in just one month without the governmental economic support. The Japanese universal financial support program [17] was associated with better health outcomes.

Most studies about the impact of crises of all kinds on inequalities -and therefore, health inequalities- have been in the long term, and there is still debate about the role of catastrophes in levelling inequalities or not [40, 41]. Health inequalities in COVID-19 incidence and mortality have been widely and consistently documented in Spain and other countries [18, 21, 42, 43] but, beyond the infectious disease, the COVID-19 crisis needs a short-to-medium term approach to understand how the Government measures and the crisis itself have impacted health inequalities. As some authors point out, although there is a consensus on the harmful effect of the crises on inequalities, exceptions occur if there is a period of welfare state expansion, improved health care and empowered people [40, 41]. Strong democratic systems with robust deliberative decision-making processes have shown to protect populations against excess deaths by all causes during the COVID-19 pandemic [44], and universal health coverage and social protection systems protect vulnerable populations worldwide [45]. Also, community participation in coping with crises is key to reduce gaps in inequalities [46]; in this sense, there were some experiences in Spain with potentially good results, although they need to be assessed [19]. Our findings suggest that the impact on inequalities might have been mitigated through the governmental economic support to low and middle education level groups months later, after the lockdown.

### Differences by education level among women and men

Relative inequalities in health were observed in women and men: PR adjusted by age showed statistically significant differences in both genders with similar magnitude. However, in the multivariate models, adjusting for all the variables, health inequalities per education level remained statistically significant among women in most dimensions just after the lockdown, while in men they were only seen in one dimension nine months later. This could be explained by the fact that women have a double vulnerability: first, although they live longer than men, they usually have poorer health, as previously found in Spain [47] and several other European countries [48]. Second, they have worse socioeconomic conditions [49]. For example, 4.2% of women with low level of education changed their status from workers to non-workers during the follow-up, while among those with high education level the change is in the opposite direction, as 4.6% changed their status from non-workers to workers. This change was negligible among men, and these gender differences in getting, keeping, or losing employment might partly explain why relative inequalities in health problems persisted statistically significant among women.

### The role of age and comorbidity

Age and comorbidity are well known determinants of health [37]. After adjusting for age, one of the main confounding variables, differences by education level decreased, but were still statistically significant in most dimensions and both surveys. Chronic health conditions had an important role in these inequalities too, since there is consistent evidence on the higher prevalence of health problems among people with lower socioeconomic resources [49]. Considering which are the most suitable estimators to quantify COVID-19 inequities in our study, age-adjusted estimators are necessary to avoid the confounding role of age, as older participants compose the low education level group. On the other hand, when adjusting for the number of chronic conditions, we adjust for the pre-pandemic health problems, so this ensures that estimators obtained are consequence of the pandemic. However, since the impact of the COVID-19 is likely worse in individuals with chronic conditions, to adjust for them may imply an over adjustment and underestimation of inequalities.

### The role of COVID-19, household and working conditions during lockdown

Living with a partner during the lockdown and shortly after was a protective factor against anxiety/depression among men, while we did not find this for women. This is in line with previous studies reporting it as a protective factor only in men [50]. Factors related to space during lockdown (having 3 or more bedrooms, or access to a garden or balcony) showed to be protective against anxiety/depression and pain/discomfort among women. This might be related to the fact that more women than men reported being homemakers, on a sick leave or not having a paid employment, which makes them be more time at home. Similar results to ours have been found in France, where wellbeing was negatively impacted by having no access to outdoors spaces or living in a small home [51], and in Portugal, where access to a garden during lockdown was protective against mental health problems [52].

Nine months after the lockdown, at the end of the third wave of the pandemic in Spain (February-March 2021), having children in care was a risk to have pain problems in both genders. Having elderly persons in care was a risk to have anxiety/depression problems in women, but a protective factor in men. The impact of the lockdown in mental health in women compared to men has also been reported in several studies [52–55]. Women sacrificed more working hours of their paid jobs than their male partners to cover care needs of children or housekeeping, and this was the case even in women who telecommuted just like their male partners [55]. Working from home is generally easier for white-collar workers, who are the main earners, but many women are support personnel of white-collar jobs (i.e., administration), so there were many more women who had to work from home while having low or middle education level and having to take care of children or home [56]. It is also possible that forced telecommuting for workers, many of them with a high education level, might have implied more hours of home confinement and a possible negative impact in certain health dimensions, as some other studies suggest [57, 58].

As expected, working status related to age (being retired) or disease (being disabled, on a sick leave) was associated with a higher risk for health problems in any of the dimensions. Unexpectedly, working conditions such as working outside the home, an essential job, and feeling unprotected were not significantly associated with any dimension of the EQ-5D-5L in the multivariate analysis and, consequently, they were not included in the final Poisson regression models.

### Limitations

One of the main limitations of our study is that the available pre-pandemic data with EQ-5D-5L results was outdated. Probably results of this 2011/12 National Health Survey were affected by the 2008 financial crisis. The Spanish population has overcome many economic and social changes in this 10-year period, so comparing our results to this outdated information might not be accurate.

Secondly, the total sample of 3,500 participants was obtained applying quotas and post-stratification weights to achieve representativeness of the non-institutionalized Spanish inhabitants in terms of age, sex and autonomous communities. However, the proportion of participants with low education level in this total sample was lower than in the Spanish population according to data from INE [32]: 27.6% vs. 34.1% in women; and 22.0% vs. 40.2% in men. In addition, the 1,500 participants that did not answer the follow-up interview had lower socioeconomic status (see Supplementary Table S1); therefore, the subsample of 2,000 participants completing both interviews was even less representative, with only 24.6% and 19.1% of women and men, respectively, with low education level. This could lead to an underestimation of health inequalities in this group. Furthermore, as we included only non-institutionalized adults, we are probably underestimating the inequalities.

Finally, although education level could not be the best indicator of socioeconomic position, it was selected for the principal analysis because it presented a lower percentage of missings than monthly income in our sample (0.10% and 7.30%, respectively).

## Conclusions

Prevalence of health problems measured with EQ-5D-5L was higher among participants with low education level and increased during the follow-up in all education groups, but the increase was higher in women and men with a high education level. Our findings suggest that, as in most crisis, health problems appeared earlier in the low education level group. The impact of the COVID-19 pandemic appeared later in participants with a high education level and was observed at the end of the third pandemic wave in Spain. Chronic health conditions were highly associated to health problems, while home-related factors during lockdown as a spacious home and access to green spaces seem to have been protective. Our hypothesis is that the economic support given by the Spanish government during the pandemic could have had a protective role on health in the low education level group, while working from home might have been a source of stress for many participants with a high level of education, and mainly for women. Further analysis on the role of the governmental economic aid given to vulnerable people and of forced telecommuting for others might shed light on this evolution.

### Electronic supplementary material

Below is the link to the electronic supplementary material.

**Supplementary table S1** **Supplementary figure S1** Age-adjusted prevalence ratios (and 95% confidence intervals) of health problems reported in each Eq. 5D5L dimension among working participants with a low level of education compared to those with a high level of education, stratified by gender. **Supplementary figure S2** Poisson regression models of having self-care problems: adjusted prevalence ratios and 95% confidence intervals compared to the reference (Ref) category. aPR for the category other working status in men could not be estimated due to low numbers **STROBE checklist**.

## Data Availability

The de-identified participant data is available as from publication and upon reasonable request from the corresponding author as long as the main objective of the data sharing request is replicating the analysis and findings as reported in this paper (without investigator support), after approval of a proposal, and with a signed data access agreement.

## References

[1] de Jesus M, Moumni Z, Sougui ZH, Biswas N, Kubicz R, Pourtau L. “Living in confinement, stopped in Time”: migrant social vulnerability, coping and Health during the COVID-19 pandemic lockdown in France. Int J Environ Res Public Health 2022 Aug 15;19(16):10084; 10.3390/ijerph191610084. 10.3390/ijerph191610084 PMC9408687 36011730

[2] di Girolamo C, Gnavi R, Landriscina T, Forni S, Falcone M, Calandrini E et al. Indirect impact of the COVID-19 pandemic and its containment measures on social inequalities in hospital utilisation in Italy. J Epidemiol Community Health (1978). 2022 Aug;76(8):707–15; 10.1136/jech-2021-218452. 10.1136/jech-2021-218452 35552241

[3] Bakaloudi DR, Jeyakumar DT, Jayawardena R, Chourdakis M (2021). The impact of COVID-19 lockdown on snacking habits, fast-food and alcohol consumption: a systematic review of the evidence. Clin Nutr.

[4] Bann D, Villadsen A, Maddock J, Hughes A, Ploubidis GB, Silverwood R (2021). Changes in the behavioural determinants of health during the COVID-19 pandemic: gender, socioeconomic and ethnic inequalities in five british cohort studies. J Epidemiol Community Health (1978).

[5] Islam N, Jdanov DA, Shkolnikov VM, Khunti K, Kawachi I, White M, et al. Effects of covid-19 pandemic on life expectancy and premature mortality in 2020: time series analysis in 37 countries. BMJ. 2021;e066768–8. 10.1136/bmj-2021-066768. 10.1136/bmj-2021-066768 PMC8564739 34732390

[6] Kaczorowski J, del Grande C (2021). Beyond the tip of the iceberg: direct and indirect effects of COVID-19. Lancet Digit Health.

[7] Wang H, Paulson KR, Pease SA, Watson S, Comfort H, Zheng P, et al. Estimating excess mortality due to the COVID-19 pandemic: a systematic analysis of COVID-19-related mortality, 2020–21. The Lancet. 2022 Apr;399(10334):1513–36. 10.1016/S0140-6736(21)02796-3. 10.1016/S0140-6736(21)02796-3 PMC8912932 35279232

[8] Real Decreto 463. /2020, de 14 de marzo, por el que se declara el estado de alarma para la gestión de la situación de crisis sanitaria ocasionada por el COVID-19. (Boletín Oficial del Estado, número 67, de 14 de marzo de 2020). https://www.boe.es/buscar/doc.php?id=BOE-A-2020-3692. Accessed 2021 Dec 1.

[9] Malina D, Rosenbaum L (2020). The untold toll-the pandemic’s Effects on patients without Covid-19. 2020. N Engl J Med.

[10] Chang AY, Cullen MR, Harrington RA, Barry M (2021). The impact of novel coronavirus COVID-19 on noncommunicable disease patients and health systems: a review. J Intern Med.

[11] Cuschieri S, Mamo J (2021). Taking care of the ordinary in extraordinary times—delayed routine care means more morbidity and pre-mature mortality. Eur J Public Health.

[12] Dorrucci M, Minelli G, Boros S, Manno V, Prati S, Battaglini M et al. Excess mortality in Italy during the COVID-19 pandemic: assessing the differences between the First and the Second Wave, Year 2020. Front Public Health 2021 Jul 16;9; 10.3389/fpubh.2021.669209. 10.3389/fpubh.2021.669209 PMC8322580 34336767

[13] Konstantinoudis G, Cameletti M, Gómez-Rubio V, Gómez IL, Pirani M, Baio G et al. Regional excess mortality during the 2020 COVID-19 pandemic in five european countries. Nat Commun 2022 Jan 25;13(1):482; https://www.nature.com/articles/s41467-022-28157-3. 10.1038/s41467-022-28157-3 PMC8789777 35079022

[14] Glover RE, van Schalkwyk MCI, Akl EA, Kristjannson E, Lotfi T, Petkovic J (2020). A framework for identifying and mitigating the equity harms of COVID-19 policy interventions. J Clin Epidemiol.

[15] González-Touya M, Stoyanova A, Urbanos-Garrido RM. Covid-19 and unmet healthcare needs of older people: did inequity arise in europe? Int J Environ Res Public Health. 2021;18(17). 10.3390/ijerph18179177. 10.3390/ijerph18179177 PMC8431067 34501767

[16] Aspachs O, Durante R, Graziano A, Mestres J, Reynal-Querol M, Montalvo JG (2021). Tracking the impact of COVID-19 on economic inequality at high frequency. PLoS ONE.

[17] Ikeda T, Igarashi A, Odani S, Murakami M, Tabuchi T (2021). Health-Related Quality of Life during COVID-19 pandemic: assessing impacts of job loss and Financial Support Programs in Japan. Appl Res Qual Life.

[18] Bacigalupe A, Martín U, Franco M, Borrell C (2022). Desigualdades socioeconómicas y COVID-19 en España. Informe SESPAS 2022. Gac Sanit.

[19] Malmusi D, Pasarín MI, Marí-Dell’Olmo M, Artazcoz L, Diez E, Tolosa S et al. Multi-level policy responses to tackle socioeconomic inequalities in the incidence of COVID-19 in a European urban area. Int J Equity Health. 2022 Dec 19;21(1):28; 10.1186/s12939-022-01628-1. 10.1186/s12939-022-01628-1 PMC8857870 35183189

[20] Marí-Dell’Olmo M, Gotsens M, Pasarín MI, Rodríguez-Sanz M, Artazcoz L, Garcia de Olalla P et al. Socioeconomic inequalities in COVID-19 in a european Urban Area: two waves, two patterns. Int J Environ Res Public Health 2021 Jan 30;18(3):1256; 10.3390/ijerph18031256. 10.3390/ijerph18031256 PMC7908269 33573323

[21] Bambra C, Riordan R, Ford J, Matthews F (2020). The COVID-19 pandemic and health inequalities. J Epidemiol Commun Health.

[22] Chen JT, Krieger N. Revealing the unequal burden of COVID-19 by income, race/ethnicity, and household crowding: US county versus zip code analyses. Journal of Public Health Management and Practice. 2021;27 Suppl 1, COVID-19 and Public Health: Looking Back, Moving Forward:S43-S56. doi: 10.1097/PHH.0000000000001263. 10.1097/PHH.0000000000001263 32956299

[23] Khanijahani A, Iezadi S, Gholipour K, Azami-Aghdash S, Naghibi D. A systematic review of racial/ethnic and socioeconomic disparities in COVID-19. Int J Equity Health 2021 Dec 24;20(1):248; 10.1186/s12939-021-01582-4. 10.1186/s12939-021-01582-4 PMC8611382 34819081

[24] O’Connor RC, Wetherall K, Cleare S, McClelland H, Melson AJ, Niedzwiedz CL (2021). Mental health and well-being during the COVID-19 pandemic: longitudinal analyses of adults in the UK COVID-19 Mental Health & Wellbeing study. Br J Psychiatry.

[25] Esteve-Matalí L, Llorens-Serrano C, Alonso J, Vilagut G, Moncada S, Navarro-Giné A. Mental health inequalities in times of crisis: evolution between 2005 and 2021 among the Spanish salaried population. J Epidemiol Community Health (1978). 2023 Jan;77(1):38–43; 10.1136/jech-2022-219523. 10.1136/jech-2022-219523 36344271

[26] Azizi A, Achak D, Aboudi K, Saad E, Nejjari C, Nouira Y (2020). Health-related quality of life and behavior-related lifestyle changes due to the COVID-19 home confinement: dataset from a moroccan sample. Data Brief.

[27] Ferreira LN, Pereira LN, da Fé Brás M, Ilchuk K (2021). Quality of life under the COVID-19 quarantine. Qual Life Res.

[28] Hay JW, Gong CL, Jiao X, Zawadzki NK, Zawadzki RS, Pickard AS (2021). A US Population Health Survey on the impact of COVID-19 using the EQ-5D-5L. J Gen Intern Med.

[29] Long D, Haagsma JA, Janssen MF, Yfantopoulos JN, Lubetkin EI, Bonsel GJ. Health-related quality of life and mental well-being of healthy and diseased persons in 8 countries: does stringency of government response against early COVID-19 matter? SSM Popul Health. 2021;15. 10.1016/j.ssmph.2021.100913. 10.1016/j.ssmph.2021.100913 PMC8426285 34522763

[30] MIND/COVID-19. : Mental health Impact and NeeDs associated with COVID-19: a comprehensive national evaluation in Spain. https://www.mindcovid.org/home. Accessed 2021 Dec 13.

[31] Mortier P, Vilagut G, Ferrer M, Alayo I, Bruffaerts R, Cristóbal-Narváez P et al. Thirty-day suicidal thoughts and behaviours in the spanish adult general population during the first wave of the Spain COVID-19 pandemic. Epidemiol Psychiatr Sci 2021 Feb 17;30:e19, 10.1017/S2045796021000093. 10.1017/S2045796021000093 PMC7925988 34187614

[32] Instituto Nacional de Estadística. https://www.ine.es/. Accessed 2021 Mar 13.

[33] Hernandez G, Garin O, Pardo Y, Vilagut G, Pont À, Suárez M (2018). Validity of the EQ–5D–5L and reference norms for the spanish population. Qual Life Res.

[34] Martí-Pastor M, Pont A, Ávila M, Garin O, Vilagut G, Forero CG (2018). Head-to-head comparison between the EQ-5D-5L and the EQ-5D-3L in general population health surveys. Popul Health Metr.

[35] International Standard Classification of Education. Unesco Institute for Statistics. 2011. http://uis.unesco.org/sites/default/files/documents/international-standard-classification-of-education-isced-2011-en.pdf. Accessed 2021 Feb 18.

[36] Zou G. A Modified Poisson Regression Approach to Prospective Studies with Binary Data. Am J Epidemiol. 2004 Apr 1;159(7):702–6.; 10.1093/aje/kwh090. 10.1093/aje/kwh090 15033648

[37] de Sanidad M. Servicios Sociales e Igualdad. Comisión para reducir las desigualdades sociales en salud en España. Avanzando hacia la equidad. Propuestas de políticas e intervenciones para reducir las desigualdades sociales en salud en España. Madrid 2015. https://www.mscbs.gob.es/profesionales/saludPublica/prevPromocion/promocion/desigualdadSalud/docs/Propuesta_Politicas_Reducir_Desigualdades.pdf. Accessed 2021 Dec 13.

[38] R Core Team. A language and environment for statistical computing. R Foundation for Statistical Computing, Vienna, Austria. 2021. https://www.R-project.org/. Accessed 2021 Dec 13.

[39] Arrospide A, Machón M, Ramos-Goñi JM, Ibarrondo O, Mar J (2019). Inequalities in health-related quality of life according to age, gender, educational level, social class, body mass index and chronic diseases using the spanish value set for Euroquol 5D-5L questionnaire. Health Qual Life Outcomes.

[40] van Bavel B, Scheffer M (2021). Historical effects of shocks on inequality: the great leveler revisited. Humanit Soc Sci Commun.

[41] Bambra C. Levelling up: global examples of reducing health inequalities. Scand J Public Health. 2021;14034948211022428. 10.1177/14034948211022428. 10.1177/14034948211022428 PMC9578091 34148458

[42] Ministerio de Sanidad. Equidad en Salud y COVID- 19. Análisis y propuestas para abordar la vulnerabilidad epidemiológica vinculada a las desigualdades sociales. Madrid. ; 2020. Available from: https://www.sanidad.gob.es/profesionales/saludPublica/ccayes/alertasActual/nCov/documentos/COVID19_Equidad_en_salud_y_COVID-19.pdf. Accessed 2023 January 30.

[43] Ordovás JM, Esteban M, García-Retamero R, Valcárcel BGL, Gordaliza A, Inzitari M et al. Informe del GTM sobre Desigualdades y Covid-19. 2021. http://hdl.handle.net/10261/239476.

[44] Jain V, Clarke J, Beaney T. Association between democratic governance and excess mortality during the COVID-19 pandemic: an observational study. J Epidemiol Community Health (1978). 2022 Oct;76(10):853–60; 10.1136/jech-2022-218920. 10.1136/jech-2022-218920 35768188

[45] Barron GC, Laryea-Adjei G, Vike-Freiberga V, Abubakar I, Dakkak H, Devakumar D et al. Safeguarding people living in vulnerable conditions in the COVID-19 era through universal health coverage and social protection. Lancet Public Health 2022 Jan;7(1):e86–92; 10.1016/S2468-2667(21)00235-8. 10.1016/S2468-2667(21)00235-8 PMC8665842 34906331

[46] Marston C, Renedo A, Miles S. Community participation is crucial in a pandemic. The Lancet. 2020 May;395(10238):1676–8; 10.1016/S0140-6736(20)31054-0. 10.1016/S0140-6736(20)31054-0 PMC7198202 32380042

[47] Solé-Auró A, Zueras P, Lozano M, Rentería E. Gender Gap in Unhealthy Life Expectancy: The Role of Education Among Adults Aged 45+. Int J Public Health. 2022 Aug 24;67; 10.3389/ijph.2022.1604946 PMC9459479 36090830

[48] European Institute for Gender Equality. Gender Equality Index. 2019. Work-life balance. 2020 [cited 2023 Jan 31]. Available from: https://eige.europa.eu/publications/gender-equality-index-2019-report/women-live-longer-poorer-health. Accessed 2023 January 28.

[49] Marmot M, Allen J, Bell R, Bloomer E, Goldblatt P (2012). WHO European review of social determinants of health and the health divide. The Lancet.

[50] Grundström J, Konttinen H, Berg N, Kiviruusu O (2021). Associations between relationship status and mental well-being in different life phases from young to middle adulthood. SSM Popul Health.

[51] Haesebaert F, Haesebaert J, Zante E, Franck N. Who maintains good mental health in a locked-down country? A french nationwide online survey of 11,391 participants. Health Place 2020 Nov;66:102440; doi: 10.1016/j.healthplace.2020.102440. 10.1016/j.healthplace.2020.102440 PMC7490637 32947185

[52] Silva Moreira P, Ferreira S, Couto B, Machado-Sousa M, Fernández M, Raposo-Lima C et al. Protective elements of Mental Health Status during the COVID-19 outbreak in the Portuguese Population. Int J Environ Res Public Health 2021 Feb 16;18(4):1910; 10.3390/ijerph18041910. 10.3390/ijerph18041910 PMC7920474 33669453

[53] Xue B, McMunn A (2021). Gender differences in unpaid care work and psychological distress in the UK Covid-19 lockdown. PLoS ONE.

[54] Qian Y, Hu Y (2021). Couples’ changing work patterns in the United Kingdom and the United States during the COVID-19 pandemic. Gend Work Organ.

[55] Collins C, Landivar LC, Ruppanner L, Scarborough WJ (2021). COVID-19 and the gender gap in work hours. Gend Work Organ.

[56] Yavorsky JE, Qian Y, Sargent AC. The gendered pandemic: The implications of COVID-19 for work and family. Sociol Compass. 2021 Jun 9;15(6):e12881; 10.1111/soc4.12881. 10.1111/soc4.12881 PMC8250288 34230836

[57] Wood SJ, Michaelides G, Inceoglu I, Hurren ET, Daniels K, Niven K, Homeworking (2021). Well-being and the COVID-19 pandemic: a Diary Study. Int J Environ Res Public Health.

[58] Galanti T, Guidetti G, Mazzei E, Zappalà S, Toscano F (2021). Work from Home during the COVID-19 outbreak: the impact on employees’ remote work Productivity, Engagement, and stress. J Occup Environ Med.

