# Correction: Social inequalities in mental and physical health derived from the COVID-19 pandemic in Spain beyond SARS-CoV-2 infection

**DOI:** 10.1186/s12939-023-01997-1

**Published:** 2023-09-19

**Authors:** Isabel Moreira, Montse Ferrer, Gemma Vilagut, Philippe Mortier, Mireia Felez-Nobrega, Joan Domènech-Abella, Josep-Maria Haro, Jordi Alonso

**Affiliations:** 1 Health Services Research Group, Hospital del Mar Research Institute, Doctor Aiguader 88, Office 144, 08003 Barcelona, Spain; 2 Preventive Medicine and Public Health Training Unit PSMar-UPF-ASPB (Parc de Salut Mar – Universitat Pompeu Fabra - Agència de Salut Pública de Barcelona), Barcelona, Spain; 3 CIBER de Epidemiología Y Salud Pública (CIBERESP), Barcelona, Spain; 4 Department of Experimental and Health Sciences, Universitat Pompeu Fabra, Barcelona, Spain; 5 Parc Sanitari Sant Joan de Déu, Barcelona, Spain; 6 CIBER de Salud Mental (CIBERSAM), Madrid, Spain; 7 Instituto de Investigación del Hospital de La Princesa, Madrid, Spain

**Correction: Int J Equity Health 22, 136 (2023)**

**https://doi.org/10.1186/s12939-023-01933-3**

After publication of this article [1], the authors reported that the wrong Supplementary file was originally published with this article; it has now been replaced with the correct file.

The original article [1] has been corrected.

### Supplementary Information

**Additional file 1: Supplementary table S1.** Socio-demographic differences between respondents of both the first and second survey (*n*=2000) and those who only answered the first one (*n*=1500). Unweighted frequencies and percentages. **Supplementary figure S1. **Age-adjusted prevalence ratios (and 95% confidence intervals) of health problems reported in each EQ5D5L dimension among working participants with a low level of education compared to those with a high level of education, stratified by gender. **Supplementary figure S2.** Poisson regression models of having self-care problems: adjusted prevalence ratios and 95% confidence intervals compared to the reference (Ref) category. aPR for the category other working status in men could not be estimated due to low numbers. **STROBE checklist.**
