# Supplementary material for: Social inequalities in mental and physical health derived from the COVID-19 pandemic in Spain beyond SARS-CoV-2 infection
Source: Int J Equity Health. 2023 Jul 24;22:136. doi: 10.1186/s12939-023-01933-3 (PMC10367254; doi:10.1186/s12939-023-01933-3)
Supplement: Supplementary file 1 — Supplementary table S1 Supplementary figure S1 Age-adjusted prevalence ratios (and 95% confidence intervals) of health problems reported in each Eq. 5D5L dimension among working participants with a low level of education compared to those with a high level of education, stratified by gender. Supplementary figure S2 Poisson regression models of having self-care problems: adjusted prevalence ratios and 95% confidence intervals compared to the reference (Ref) category. aPR for the category other working status in men could not be estimated due to low numbers STROBE checklist. [file 12939_2023_1933_MOESM1_ESM.pdf]

SOCIAL INEQUALITIES IN MENTAL AND PHYSICAL HEALTH DERIVED FROM THE COVID-19 PANDEMIC IN SPAIN BEYOND SARS-CoV-2 INFECTION

International Journal for Equity in Health

Isabel Moreira[1,2], Montse Ferrer[1,3,4]*, Gemma Vilagut[1,3], Philippe Mortier[1,3], Mireia Felez-Nobrega[5], Joan Domènech-Abella[5,6], Josep-Maria Haro[5,6,7†] and Jordi Alonso[1,3,4†]

[1]Health Services Research Group, Hospital del Mar Research Institute, Barcelona, Spain

[2] Preventive Medicine and Public Health Training Unit PSMar-UPF-ASPB (Parc de Salut Mar – Universitat Pompeu Fabra - Agència de Salut Pública de Barcelona), Barcelona, Spain

[3]CIBER de Epidemiología y Salud Pública (CIBERESP), Spain

[4] Department of Experimental and Health Sciences, Universitat Pompeu Fabra, Barcelona, Spain

[5] Parc Sanitari Sant Joan de Déu, Barcelona, Spain

[6] CIBER de Salud Mental (CIBERSAM), Madrid, Spain

[7] Instituto de Investigación del Hospital de la Princesa, Madrid, Spain

† Equal senior authors

**Corresponding author:*mferrer@imim.es

Content of the document:

Supplementary table S1

Supplementary figure S1. Age-adjusted prevalence ratios (and 95% confidence intervals) of health problems reported in each EQ5D5L dimension among working participants with a low level of education compared to those with a high level of education, stratified by gender

Supplementary figure S2. Poisson regression models of having self-care problems: adjusted prevalence ratios and 95% confidence intervals compared to the reference (Ref) category. aPR for the category other working status in men could not be estimated due to low numbers

STROBE checklist

Supplementary table S1 Socio-demographic differences between respondents of both the first and second survey (n=2000) and those who only answered the first one (n=1500). Unweighted frequencies and percentages

	First and second survey (n=2000)	Only first survey (n=1500)	Unweighted p-value
**SOCIODEMOGRAPHIC**			
**Age group**			< 0.001*
18 - 34 years	365 (18.25 %)	332 (22.13 %)	
35 - 54 years	869 (43.45 %)	632 (42.13 %)	
55 – 64 years	430 (21.50 %)	250 (16.67 %)	
65 years and over	336 (16.80 %)	286 (19.07 %)	
**Education level**			<0.001*
Secondary education or lower	433 (21.67 %)	447 (29.82 %)	
Baccalaureate to undergraduate	679 (33.98 %)	532 (35.49 %)	
University degree or higher	886 (44.34 %)	520 (34.69 %)	
Missings	*2 (0.10 %)*	*1 (0.07 %)*	
**Country of birth**			0.002*
Spain	1768 (88.40 %)	1273 (84.87 %)	
Abroad	232 (11.60 %)	227 (15.13 %)	
**Country of birth if born abroad**			0.223
Low income countries	36 (15.58 %)	41 (19.71 %)	
Middle income countries	149 (64.50 %)	137 (65.87 %)	
High income countries	46 (19.91 %)	30 (14.42 %)	
Missings	*1 (0.43 %)*	*19 (8.37%)*	
**Monthly income**			<0.001*
< 1050 €	392 (21.14 %)	352 (26.55 %)	
1050 to < 2700 €	1029 (55.50 %)	726 (54.75 %)	
≥ 2700 €	433 (23.35 %)	248 (18.70 %)	
Missings	*146 (7.30 %)*	*174 (11.60 %)*	
**NUMBER OF CHRONIC HEALTH CONDITIONS**			0.184
0	1208 (60.49 %)	911 (60.77 %)	
1	563 (28.18 %)	398 (26.56 %)	
2	174 (8.71 %)	132 (8.81 %)	
3 or more	53 (2.65 %)	58 (3.87 %)	
Missings	2 (0.10 %)	1 (0.07 %)	
**COVID AND LOCKDOWN**			
**COVID-19: Personal infection**			0.305
Negative	1939 (97.19 %)	1458 (97.59 %)	
Positive or COVID diagnosis, non-severe	53 (2.66 %)	31 (2.07 %)	
Hospitalized	3 (0.15 %)	5 (0.33 %)	
Missings	*5 (0.25 %)*	*6 (0.40 %)*	
**COVID-19: Having a beloved one infected**			0.007*
No	1027 (51.89 %)	840 (57.18 %)	
Yes, but not inner circle	879 (44.42 %)	575 (39.14 %)	
Yes, inner circle	73 (3.69 %)	54 (3.68 %)	
Missings	*21 (1.05 %)*	*31 (2.07 %)*	
**Living with a partner**			0.037*
No	672 (33.60 %)	556 (37.07 %)	
Yes	1328 (66.40 %)	944 (62.93 %)	
**Having children in care**			0.875
No	1319 (65.95 %)	984 (65.60 %)	
Yes	681 (34.05 %)	516 (34.40 %)	
**Having elderly people or people with a disability in care**			0.138
No	1705 (85.25 %)	1306 (87.07 %)	

Yes	295 (14.75 %)	194 (12.93 %)	
**Number of bedrooms**			0.110
0 to 3 bedrooms	1324 (66.40 %)	1023 (69.03 %)	
More than 3 bedrooms	670 (33.60 %)	459 (30.97 %)	
Missings	*6 (0.30 %)*	*18 (1.20 %)*	
**Has access to balcony or private garden**			0.347
No	567 (28.38 %)	44 (29.90 %)	
Yes	1431 (71.62 %)	1041 (70.10 %)	
Missings	*2 (0.10 %)*	*15 (1.00 %)*	
**Working status**			0.295
Working	1007 (50.55 %)	698 (46.69 %)	
Working but sick leave	54 (2.71 %)	37 (2.47 %)	
Unemployed	337 (16.92 %)	282 (18.86 %)	
Homemaker	99 (4.97 %)	95 (6.35 %)	
Student	73 (3.66 %)	60 (4.01 %)	
Disabled	54 (2.71 %)	38 (2.54 %)	
Retired	357 (17.92 %)	274 (18.33 %)	
Other	11 (0.55 %)	11 (0.74 %)	
Missings	*8 (0.40 %)*	*5 (0.33 %)*	
**Working conditions (only for workers)**	N= 1007	N= 698	
**Frequency of working outside home**			0.006*
Never	432 (42.99 %)	280 (40.35 %)	
Rarely	119 (11.84 %)	53 (7.64 %)	
Sometimes	76 (7.56 %)	71 (10.23 %)	
Frequently	33 (3.28 %)	27 (3.89 %)	
Usually	68 (6.77 %)	37 (5.33 %)	
Always	277 (27.56 %)	226 (32.56 %)	
Missings	*2 (0.20 %)*	*4 (0.57 %)*	
**Job considered as essential**			0.195
Yes	528 (53.55 %)	393 (57.79 %)	
No	458 (46.45 %)	287 (42.21 %)	
Missings	*21 (2.09 %)*	*18 (2.58 %)*	
**Frequency of feeling unprotected against SARS-CoV-2 at work**			0.109
Never	559 (55.62 %)	348 (50.00 %)	
Rarely	134 (13.33 %)	100 (14.37 %)	
Sometimes	141 (14.03 %)	114 (16.38 %)	
Usually	53 (5.27 %)	55 (7.90 %)	
Always	111 (11.04 %)	74 (10.63 %)	
Doesn’t know	7 (0.70 %)	5 (0.72 %)	
Missings	*2 (0.20 %)*	*2 (0.29 %)*	

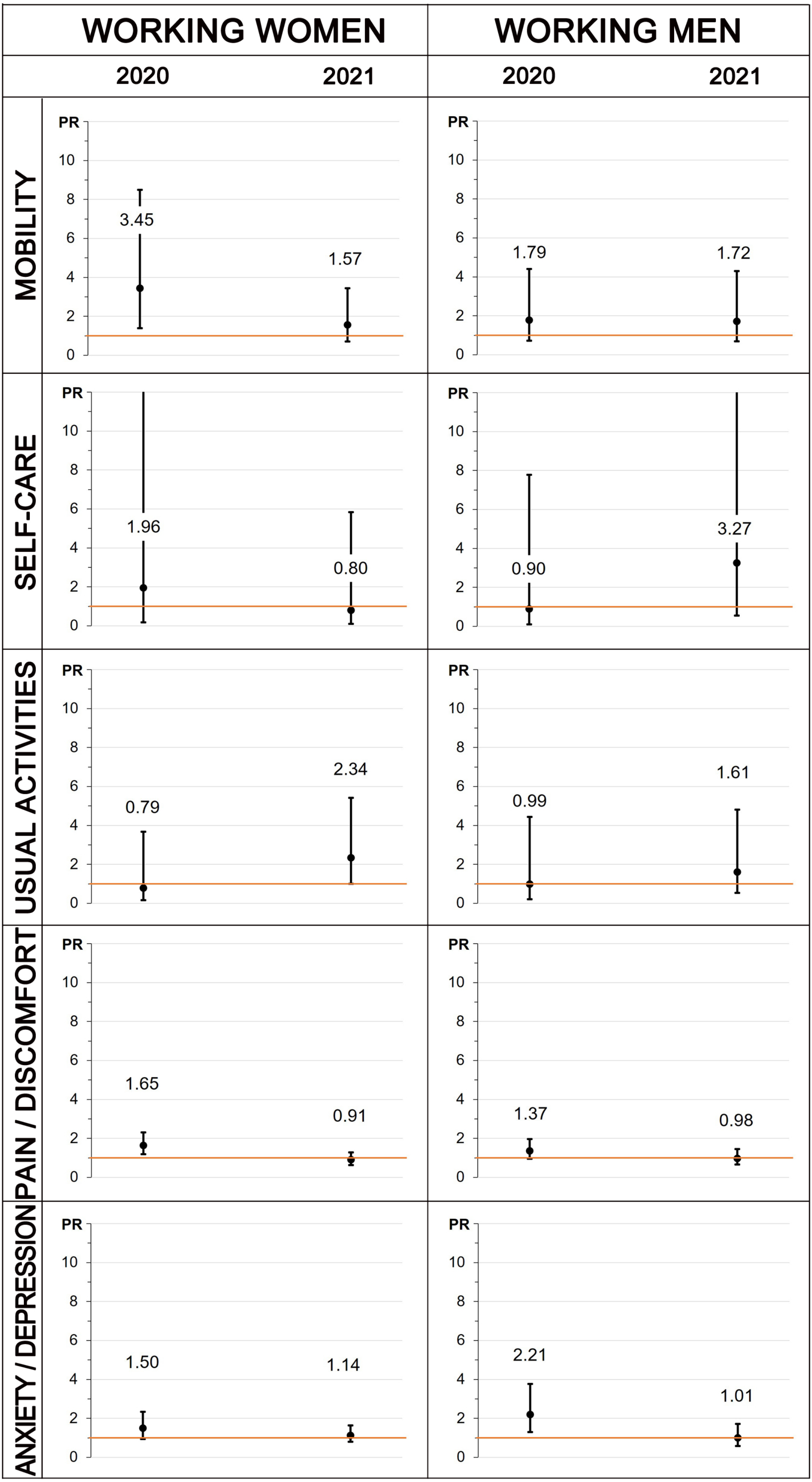
WORKING WOMEN
WORKING MEN
2020
2021
2020
2021
MOBILITY
SELF-CARE
USUAL ACTIVITIES
PAIN / DISCOMFORT
ANXIETY / DEPRESSION
PR
0
2
4
6
8
10
3.45
1.57
1.79
1.72
1.96
0.80
0.90
3.27
0.79
2.34
0.99
1.61
1.65
0.91
1.37
0.98
1.50
1.14
2.21
1.01

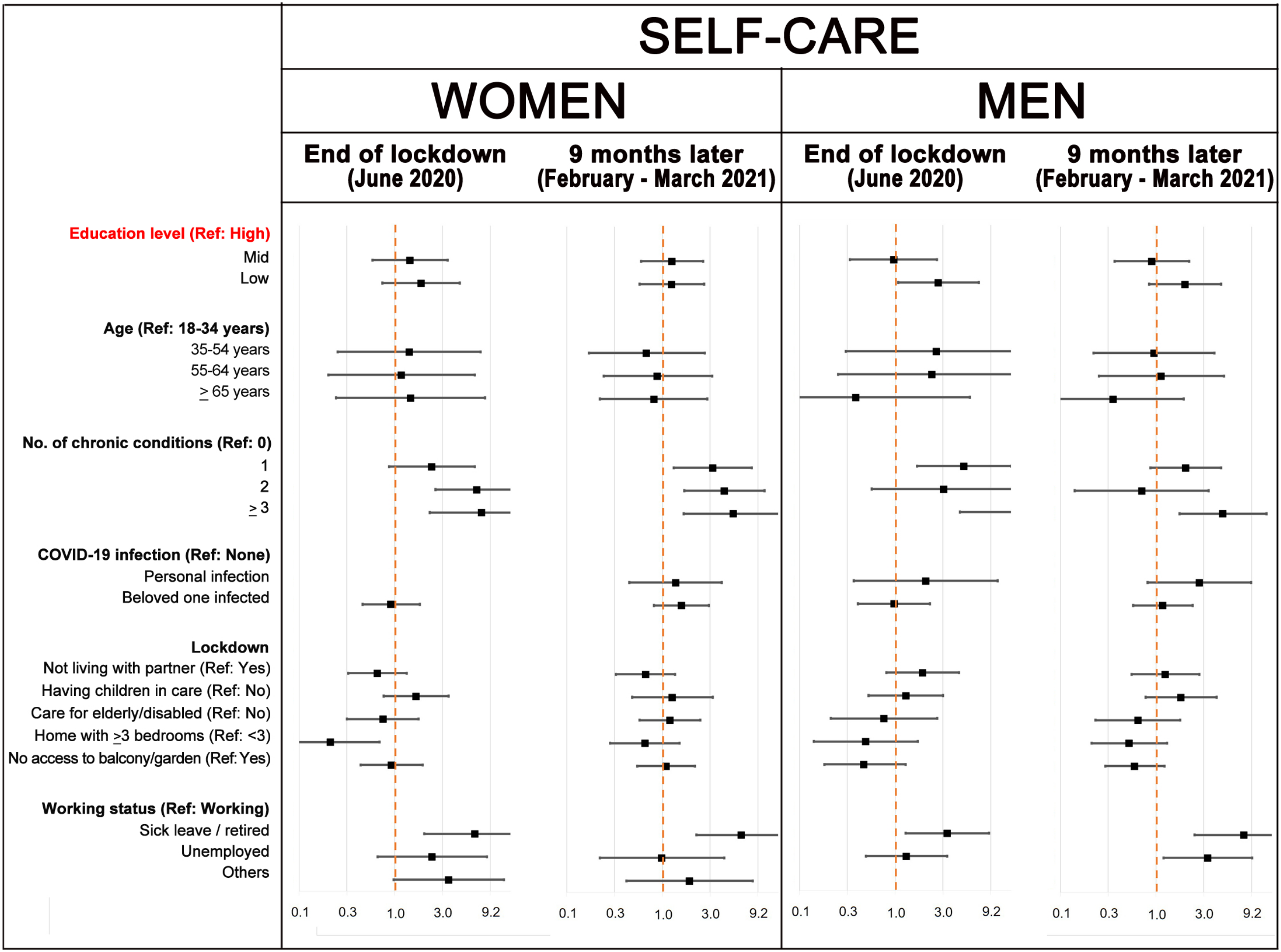
SELF-CARE
WOMEN
MEN
End of lockdown (June 2020)
9 months later (February - March 2021)
End of lockdown (June 2020)
9 months later (February - March 2021)
Education level (Ref: High)
Mid
Low
Age (Ref: 18-34 years)
35-54 years
55-64 years
≥ 65 years
No. of chronic conditions (Ref: 0)
1
2
≥ 3
COVID-19 infection (Ref: None)
Personal infection
Beloved one infected
Lockdown
Not living with partner (Ref: Yes)
Having children in care (Ref: No)
Care for elderly/disabled (Ref: No)
Home with ≥3 bedrooms (Ref: <3)
No access to balcony/garden (Ref: Yes)
Working status (Ref: Working)
Sick leave / retired
Unemployed
Others
0.1
0.3
1.0
3.0
9.2

STROBE Statement—checklist of items that should be included in reports of observational studies

	Item No	Recommendation	Page No
**Title and abstract**	1	(*a*) Indicate the study's design with a commonly used term in the title or the abstract	2
		(*b*) Provide in the abstract an informative and balanced summary of what was done and what was found	2
**Introduction**			
Background/rationale	2	Explain the scientific background and rationale for the investigation being reported	3-4
Objectives	3	State specific objectives, including any prespecified hypotheses	4
**Methods**			
Study design	4	Present key elements of study design early in the paper	4-5
Setting	5	Describe the setting, locations, and relevant dates, including periods of recruitment, exposure, follow-up, and data collection	5
Participants	6	(*a*) *Cohort study*—Give the eligibility criteria, and the sources and methods of selection of participants. Describe methods of follow-up<br>*Case-control study*—Give the eligibility criteria, and the sources and methods of case ascertainment and control selection. Give the rationale for the choice of cases and controls<br>*Cross-sectional study*—Give the eligibility criteria, and the sources and methods of selection of participants	5
		(*b*) *Cohort study*—For matched studies, give matching criteria and number of exposed and unexposed<br>*Case-control study*—For matched studies, give matching criteria and the number of controls per case	
Variables	7	Clearly define all outcomes, exposures, predictors, potential confounders, and effect modifiers. Give diagnostic criteria, if applicable	5-6
Data sources/ measurement	8*	For each variable of interest, give sources of data and details of methods of assessment (measurement). Describe comparability of assessment methods if there is more than one group	5-7
Bias	9	Describe any efforts to address potential sources of bias	7
Study size	10	Explain how the study size was arrived at	5-6
Quantitative variables	11	Explain how quantitative variables were handled in the analyses. If applicable, describe which groupings were chosen and why	6-8
Statistical methods	12	(*a*) Describe all statistical methods, including those used to control for confounding	7-8
		(*b*) Describe any methods used to examine subgroups and interactions	7-8
		(*c*) Explain how missing data were addressed	7
		(*d*) *Cohort study*—If applicable, explain how loss to follow-up was addressed<br>*Case-control study*—If applicable, explain how matching of cases and controls was addressed<br>*Cross-sectional study*—If applicable, describe analytical methods taking account of sampling strategy	7
		(*e*) Describe any sensitivity analyses	7-8

Continued on next page

**Results**			
Participants	13*	(a) Report numbers of individuals at each stage of study—eg numbers potentially eligible, examined for eligibility, confirmed eligible, included in the study, completing follow-up, and analysed	8
		(b) Give reasons for non-participation at each stage	5
		(c) Consider use of a flow diagram	
Descriptive data	14*	(a) Give characteristics of study participants (eg demographic, clinical, social) and information on exposures and potential confounders	8
		(b) Indicate number of participants with missing data for each variable of interest	8 (Table 1)
		(c) *Cohort study*—Summarise follow-up time (eg, average and total amount)	6
Outcome data	15*	*Cohort study*—Report numbers of outcome events or summary measures over time	
		Case-control study—Report numbers in each exposure category, or summary measures of exposure	
		Cross-sectional study—Report numbers of outcome events or summary measures	8-9
Main results	16	(*a*) Give unadjusted estimates and, if applicable, confounder-adjusted estimates and their precision (eg, 95% confidence interval). Make clear which confounders were adjusted for and why they were included	
		(*b*) Report category boundaries when continuous variables were categorized	
		(*c*) If relevant, consider translating estimates of relative risk into absolute risk for a meaningful time period	9
Other analyses	17	Report other analyses done—eg analyses of subgroups and interactions, and sensitivity analyses	10
**Discussion**			
Key results	18	Summarise key results with reference to study objectives	10-14
Limitations	19	Discuss limitations of the study, taking into account sources of potential bias or imprecision. Discuss both direction and magnitude of any potential bias	14
Interpretation	20	Give a cautious overall interpretation of results considering objectives, limitations, multiplicity of analyses, results from similar studies, and other relevant evidence	15
Generalisability	21	Discuss the generalisability (external validity) of the study results	15
**Other information**			
Funding	22	Give the source of funding and the role of the funders for the present study and, if applicable, for the original study on which the present article is based	16

*Give information separately for cases and controls in case-control studies and, if applicable, for exposed and unexposed groups in cohort and cross-sectional studies.

Note: An Explanation and Elaboration article discusses each checklist item and gives methodological background and published examples of transparent reporting. The STROBE checklist is best used in conjunction with this article (freely available on the Web sites of PLoS Medicine at http://www.plosmedicine.org/, Annals of Internal Medicine at http://www.annals.org/, and Epidemiology at http://www.epidem.com/). Information on the STROBE Initiative is available at www.strobe-statement.org.
